# Poisoning by Hydrocyanic Acid

**Published:** 1845-06

**Authors:** 


*Poisoning by Hydrocyanic Acid.*—Mr. Godfrey has published, in the *Provincial Medical Journal*, a case of poisoning by prussic acid, which involves some points of interest, not from the symptoms presented, but from the time which must have elapsed, after the imbibiton of the poison, before death occurred; and also on account of the various acts of volition performed by the suicide after he had swallowed the acid. It appears that, on the morning when he committed suicide, one of his daughters accompanied him to his office, where they were seen standing together; he sent her away with a message, and, after taking off his great-coat, proceeded to a room up stairs. After a short interval, he was seen to walk hastily out of the house, in the direction of the druggist's shop. He must have swallowed the poison—about half an ounce of prussic acid—when up stairs, for the bottle was found in the fire-place the following morning, the stopper on the table. It is presumed that he took the acid before placing the bottle on the fire, as no glass or similar utensil was found in the room; he must then have gone to the head of the stairs, a distance of ten average paces, descended the stairs, seventeen in number, proceeded to the druggist's shop, forty-five paces—realizing a total of fifty-five paces and seventeen stairs. He entered the shop in his usual manner, which was slow and steady: the druggist (a personal friend) asked him how he did? He replied in his usual tone of voice, "I want some more of that prussic acid." The druggist passed round the side and end of his counter to speak to him, and then perceived that he was in the act of placing his hand upon him, as if for support, his eyes being fixed npon him with a stare. The druggist said to him, "You have been taking the prussic acid?" to which no answer was made. He was then placed in a chair, but fell to the ground and died soon after Mr. Godfrey's arrival. Although the facts above detailed are highly interesting and important in a medico-legal point of view, inasmuch as the suicide might avail himself of his knowledge to conceal his act, or an ingenious counsel might take advantage of it to confuse the medical witness, and thus obtain the murderer's acquittal, yet it is somewhat imperfect in its details. A knowledge of the amount of anhydrous acid contained in the preparation taken by the suicide, would add greatly to the value of the facts as the character of the effect produced might then be more correctly estimated.—*Ibid.*

